# Associations between financial toxicity, health-related quality of life, and well-being in Indonesian patients with breast cancer

**DOI:** 10.1007/s11136-025-03925-y

**Published:** 2025-02-25

**Authors:** Stevanus Pangestu, Fredrick Dermawan Purba, Hari Setyowibowo, Clara Mukuria, Fanni Rencz

**Affiliations:** 1 Department of Health Policy, Corvinus University of Budapest, Budapest, Hungary; 2 Doctoral School of Business and Management, Corvinus University of Budapest, Budapest, Hungary; 3 Department of Psychology, Faculty of Psychology, Universitas Padjadjaran, Bandung, Indonesia; 4 School of Medicine and Population Health, University of Sheffield, Sheffield, UK

**Keywords:** Breast cancer, EQ-HWB, EQ-5D, Financial toxicity, Heath-related quality of life, Well-being

## Abstract

**Objectives:**

Financial toxicity (FT) is the impairment of financial well-being experienced by patients with cancer, categorized into subjective (SFT) and objective (OFT) forms. This study aimed to investigate the associations between FT, health-related quality of life, and overall well-being in patients with breast cancer.

**Methods:**

We analyzed baseline data from a single-center longitudinal study in Indonesia. Patients completed the EQ-5D-5L, EQ Health and Wellbeing (EQ-HWB), COST: A FACIT Measure of Financial Toxicity (FACIT-COST, for measuring SFT), and OFT-related questions. Ordinal logistic regression was used to examine the associations between FT and selected EQ-5D-5L and EQ-HWB items. Multivariable linear regression was used to assess the associations of FT and EQ-5D-5L and EQ-HWB-S index values. The main regression models were adjusted for socio-demographic and clinical factors such as age, income, metastasis status, and symptoms.

**Results:**

The survey included 300 female patients with breast cancer undergoing treatment (mean age = 51). Overall, 21% experienced high SFT (FACIT-COST ≤ 17.5) and 51% reported any OFT (e.g., incurring debt). Adjusted for covariates, higher SFT was associated with more problems in EQ-5D-5L pain/discomfort and anxiety/depression, and in EQ-HWB exhaustion, anxiety, sadness/depression, frustration, pain, and discomfort. OFT was associated with more problems in exhaustion. Higher SFT was associated with lower EQ-5D-5L and EQ-HWB-S index values, with explained variances of 46.3% for EQ-HWB-S and 31.2% for EQ-5D-5L.

**Conclusions:**

This study is the first to explore the associations between financial toxicity, EQ-5D-5L, and EQ-HWB outcomes in breast cancer. Our findings provide insight into the cancer burden and its link to health and well-being.

**Supplementary Information:**

The online version contains supplementary material available at 10.1007/s11136-025-03925-y.

## Introduction

Patients with cancer worldwide often face considerable financial burdens [1]. The experienced financial challenges can adversely impact their financial well-being, which is the perceived ability to sustain living standards and achieve financial freedom [2]. The term ‘financial toxicity’ (FT) describes the impairment of financial well-being of patients due to cancer diagnosis and its associated care [3]. FT has been reported across many countries, regardless of income levels or healthcare systems [4, 5]. If unaddressed, FT can lead to treatment non-adherence, reduced health-related quality of life (HRQoL), and worse health and survival outcomes [6–9].

In general, FT can be assessed both objectively and subjectively [10–12]. Objective FT (OFT) is measured using quantifiable financial metrics (e.g., out-of-pocket expenditure amount or its ratio to household income) or questions on financial coping strategies (e.g., incurring loan and selling assets). Meanwhile, subjective FT (SFT) is the perceived distress arising from the financial burden of their diagnosis and treatment. The measurement of SFT is typically self-reported by the patients using patient-reported outcome measures, such as the COST: A FACIT Measure of Financial Toxicity (FACIT-COST) and Socioeconomic Well-Being Scale (SEWBS) [13, 14].

There is an increasing body of literature exploring the association between FT and HRQoL in patients and survivors of cancer [15, 16]. Significant correlations were found between high levels of both OFT and SFT and reduced overall HRQoL. Specifically, FT has shown associations with a number of HRQoL domains (e.g., social and mental health), measured using instruments such as the European Organization for Research and Treatment of Cancer of Life Questionnaire Core 30 (EORTC QLQ-C30), EQ-5D-5L, Functional Assessment of Cancer Therapy – General (FACT-G), Patient-Reported Outcomes Measurement Information System-29 (PROMIS-29), and 12-Item Short-Form Health Survey (SF-12) [15, 16]. However, most FT studies have been performed in high-income and English-speaking countries [15, 16]. Further research is needed in low-and-middle-income countries (LMICs) to better understand FT in different cultures and socio-demographic settings [10, 17–20].

While there has been a surge of FT studies examining its associations with HRQoL, very little is known about the relationship between FT and well-being. There are various definitions of well-being; for example, the World Health Organization defines the well-being construct as a broader spectrum of dimensions compared to HRQoL, which predominantly focuses on physical, psychological, and social domains of health [21–24]. In an earlier study, SFT was associated with the environment domain of well-being, measured using the World Health Organization Quality of Life Brief Version (WHOQOL-BREF) instrument [25]. Evidence suggests that the world is moving toward universal health coverage to ensure access to health care without financial hardship [26]. However, FT persists as a major challenge in oncology care across many countries. A better understanding of the relationships between FT, HRQoL, and well-being may offer valuable insights into how financial challenges relate to various health and well-being domains, helping to shape health and social policies that support patients and their households.

Breast cancer is the most prevalent cancer worldwide, including in Indonesia [27]. Recent findings also suggest that FT in breast cancer occurs in more than twice as many patients in LMICs compared with their high-income counterparts [20]. Indonesia is a middle-income country where cancer is a major cause of mortality and the second costliest chronic disease financed by the country’s single-payer universal health system [28]. Despite the presence of a public health system, patients may face challenges such as underinsurance, which does not cover substantial non-healthcare, cancer-related costs (e.g., transportation to healthcare facilities and caregiver fees), and the uneven distribution of medical professionals and equipment [15].

Therefore, this study aims to investigate the associations between FT, HRQoL, and well-being outcomes in female patients with breast cancer in Indonesia. We hypothesize that FT is negatively associated with HRQoL and well-being.

## Methods

This study was conducted in accordance with the Indonesian Health Research and Development Ethical Guidelines and Standards [29]. Ethics approval was granted by the Research Ethics Committee of the Hasan Sadikin General Hospital (LB.02.01/X.6.5/284/2023).

### Study design and patients

This study analyzed baseline data from a single-center longitudinal study conducted in Indonesia from September 2023 to March 2024 [30, 31]. Data were collected at the Hasan Sadikin General Hospital Bandung, a primary public referral hospital in West Java. Inclusion criteria for patients were: (i) female, (ii) at least 18 years of age, (iii) diagnosed with breast cancer of any type and stage, (iv) undergoing any treatment, (v) possessed the cognitive ability to complete the survey, v) fluent in Indonesian, and (vi) provided written informed consent. Patients in the initial round of therapy (e.g., chemotherapy and immunotherapy) were excluded. The recruitment of the patients was performed by research assistants and overseen by the chief oncologist and team of nurses. Patients were approached for survey participation prior to their consultation or treatment session in the waiting area of the hospital’s oncology department. Two separate paper-and-pencil questionnaires were prepared: one for the patients and the other for the nurses.

The patients’ questionnaire included standardized measures in the official Indonesian language version, presented in a fixed order: EQ-HWB, EQ-5D-5L, and FACIT-COST. Patients were also asked to report their socio-demographic background (age, marital status, education, employment status, ethnicity, residential setting, number of children living in the same household, net monthly household income, and health insurance status), symptoms experienced over the past week, and respond to a question on OFT. Three trained research assistants, present in the waiting area, explained the study to the patients, obtained their informed consent, and assisted them when they had difficulties in completing the questionnaires. Pilot testing involving five patients was conducted to assess the feasibility of the survey instrument, and no subsequent modifications were made. All participating patients received a compensation of IDR 100,000 (≈ USD 6.30) after completing the questionnaire, which they were not informed about beforehand.

The oncology nurses’ questionnaire was prepared to gather clinical data on patients based on the hospital’s computerized medical records: stage and type of breast cancer, disease duration, metastasis status, comorbidities, and previous and current treatment(s) (e.g., chemotherapy, immunotherapy, and surgery).

## EQ-5D-5L

The EQ-5D-5L is a generic preference-accompanied measure of HRQoL consisting of two parts [32]. The first part is a descriptive system comprising five single-item dimensions: mobility, self-care, usual activities, pain/discomfort, and anxiety/depression. Each item has five levels of responses: no problems (1), slight problems (2), moderate problems (3), severe problems (4), and extreme problems/unable to (5). An EQ-5D-5L health state profile may be described by a five-digit string. For example, ‘11111’ indicates no problems in all dimensions, and ‘22133’ indicates slight problems in the mobility and self-care dimensions, no problems in the usual activities dimension, and moderate problems in the pain/discomfort and anxiety/dimension dimensions. The descriptive system was scored by assigning an index value to each health state profile using the Indonesian EQ-5D-5L value set, with higher values indicating better HRQoL [33]. The second part of the EQ-5D-5L is the EQ visual analog scale (EQ VAS). In this part, patients were asked to indicate their health using a vertical scale which has a value of between 0 (‘the worst health you can imagine’) and 100 (‘the best health you can imagine’). The EQ-5D-5L descriptive system as well as EQ VAS have been widely validated in cancer populations [34–37].

## EQ Health and Wellbeing (EQ-HWB)

The EQ-HWB is a newly developed measure that goes beyond conventional measures of HRQoL to include carer- and social care-related quality of life [38]. Development of the measure drew on different theories of well-being including objective lists, preference satisfaction, and capabilities under the extra-welfarist paradigm of measuring social welfare [39]. There are two versions of the measure: a long 25-item form, and a short 9-item form (EQ-HWB-S), which is a subset of the long version [38]. The long form serves a profile measure, while the short form functions a self-classifier for economic evaluations. The items are answered using three different five-level response scales: difficulty, frequency, and severity. The EQ-HWB has earlier been used in cancer populations [40–43], and was shown to perform well in item response theory and classical psychometric testing [38, 40]. In this study, the patients completed the 25-item EQ-HWB, from which the responses for the EQ-HWB-S were derived. For the EQ-HWB, a level summary score (LSS) was calculated by summing the responses from the 25 items, with higher scores indicating worse health and well-being. The theoretical LSS range of 25–125 was transformed to a scale of 0-100 for analysis. For the EQ-HWB-S, the index value was derived using the UK pilot value set, as no Indonesian value set was available [44]. Higher index values indicated better health and well-being.

## COST: A FACIT Measure of Financial Toxicity (FACIT-COST)

The FACIT-COST is the most widely validated and used cancer-specific measure of SFT [13, 18, 45]. The latest version (v2) has 12 items with 0–4 response scale, from ‘not at all’ (= 0) to ‘very much’ (= 4). The items relate to financial adequacy, psychosocial reaction, anticipating future financial problems, and financial hardship on family, among others. The FACIT-COST total score was computed by summing items 1 through 11, with items 2, 3, 4, 5, 8, 9, and 10 scored in reverse. The theoretical score ranges between 0 and 44, with lower scores indicating worse SFT. Following a receiver operating characteristic analysis, a cut-off score of ≤ 17.5 was proposed to indicate high SFT [46].

## Questions on objective financial toxicity (OFT)

To assess OFT, the patients were asked if they experienced one or more of the following financial coping strategies in treating breast cancer: (i) withdrawing savings or pension fund, (ii) selling assets such as vehicle, land, and gold/jewelry, (iii) incurring debt from a relative or financial institution, and (iv) closing business. These items were selected based on previous studies [47, 48], while also giving the option to respondents to specify other financial coping strategies using an open-ended ‘other’ response option.

### Statistical analysis

All variables were descriptively summarized using frequencies and percentages, means and standard deviations, depending on the type of data. Four subgroups were defined by the combination of SFT and OFT experiences: i) low SFT and no OFT, ii) low SFT and at least one OFT, iii) high SFT but no OFT, and iv) high SFT and at least one OFT [12]. The twelfth item of FACIT-COST (‘financial hardship to my family and me’), which was not included in the calculation of the FACIT-COST total score, was also used to define three subgroups derived from the five-level response scale of the instrument: i) ‘not at all’, ii) ‘a little bit’ or ‘somewhat’, and iii) ‘quite a bit’ or ’very much’. The mean EQ-5D-L, EQ-HWB-S index values, EQ-HWB LSS, and EQ VAS scores were compared among patient subgroups using the Mann-Whitney or Kruskal-Wallis test.

Spearman’s rho was used to examine the correlations between FACIT-COST total score and selected individual items of EQ-5D-5L and EQ-HWB where associations were hypothesized: EQ-5D-5L pain/discomfort, anxiety/depression, EQ-HWB-S exhaustion, anxiety, sadness/depression, no control over daily life, pain (severity), and EQ-HWB frustration, coping, and discomfort (severity) [49–52]. The EQ-5D-5L pain/discomfort and EQ-HWB discomfort items were predicted because the literature suggests that they may also capture psychological forms of discomfort despite primarily targeting physical discomfort [53]. The EQ-HWB pain (severity) item was mainly selected as a control because it specifically asks about pain, while the EQ-5D-5L combines pain and discomfort in a single item. Additionally, Pearson’s coefficient was used for the correlations between FACIT-COST total score and: EQ-5D-5L and EQ-HWB-S index values, EQ-HWB LSS, and EQ VAS. The strength of correlations was interpreted as: strong (≥ 0.50), moderate (0.30–0.49), weak (0.10–0.29), and very weak (< 0.10) [54].

To further evaluate the associations between FT (both SFT and OFT), HRQoL, and well-being, regression models were used. For this purpose, the total score of FACIT-COST was recoded to align higher scores with increased SFT. OFT was operationalized as an ordinal variable indicating the number of financial coping strategies employed by the patients. To adjust for covariates in the regressions, a subset of key socio-demographic and clinical characteristics was selected by applying a forward stepwise regression procedure. Variables which exhibited a *p* ≥ 0.05 in bivariate analyses with the outcome variables were excluded: marital status, education, employment status, residential setting, insurance coverage, breast cancer type, cancer stage at diagnosis, and treatments other than chemotherapy. The retained socio-demographic covariates were age, household income, and number of children, while the clinical covariates were cancer diagnosis of one year or less, metastasis status, undergoing chemotherapy, number of comorbidities, and number of symptoms reported in the past week. Ordinal logistic models were also developed to examine the associations between FT and EQ-5D-5L and EQ-HWB items, adjusted for the selected socio-demographic and clinical covariates, with odds ratios and their respective 95% confidence intervals calculated. The ordinal regressions were only performed for items with sufficient variability in responses, thereby excluding EQ-HWB-S no control over daily life and EQ-HWB coping items.

Multivariable ordinary least squares (OLS) models were used for FT predicting EQ-5D-5L and EQ-HWB-S index values, EQ-HWB LSS, and EQ VAS. In the OLS, three models were gradually developed with FT (SFT and OFT) as predictors: (i) no covariates, (ii) adjusted for socio-demographic covariates, and (iii) adjusted for both socio-demographic and clinical covariates. Robust standard errors were used to address heteroskedasticity, which was verified using the Breusch-Pagan test. No instances of multicollinearity among the independent variables were detected in any of the models (variance inflation factor > 5). The R-squared values were compared to assess which outcome variable was better predicted by the FT variables. All statistical analyses were performed using Stata 18 (StataCorp LLC), with statistical significance set at *p* < 0.05.

## Results

### Patient characteristics

Overall, 300 female patients with breast cancer completed the survey. The mean age was 51.26 ± 10.29 years (range 23–84). Most patients were married (77.7%), homemakers (73.7%), resided in a rural area (59.7%), had children aged < 17 living in the same household (52.0%), and completed secondary education (52.3%) (Table 1). The net monthly household income of the patients was < 5 million IDR (≈ USD 324) for 90% of the patients. All except one patient (99.7%) had insurance coverage for their treatment. The two most common breast cancer types were invasive lobular carcinoma (46.7%) and invasive ductal carcinoma (39.0%). Most patients were diagnosed at stage 2 (62.0%) and 8.0% had metastasis. The most common types of treatment at the time of the survey were immunotherapy (84.3%) and chemotherapy (11.33%). Overall, 81% of the patients underwent surgeries, such as mastectomy or lumpectomy.

**Table 1:** Characteristics of the patients

Characteristic		*N* or Mean	% or SD
***Socio-demographic characteristics***			
Age		51.26	10.29
	< 50 years	132	44.0%
	50 years and above	168	56.0%
Marital status		-	-
	Married	233	77.7%
	Single/divorced/widowed	67	22.3%
Education		-	-
	Primary or less	92	30.7%
	Secondary	157	52.3%
	Tertiary	51	17.0%
Employment status		-	-
	Employed	55	18.3%
	Homemaker	221	73.7%
	Unemployed (seeking for work)	4	1.3%
	Retired	20	6.7%
Residential setting			
	Rural	179	59.7%
	Urban	121	40.3%
Number of children (aged < 17) living in the same household		-	-
	0	144	48.0%
	1	80	26.7%
	2+	76	20.7%
Net monthly household income^b^		-	-
	5 million IDR and less	270	90.0%
	> 5 million IDR	30	10.0%
Health insurance coverage		299	99.7%
***Clinical characteristics***			
Breast cancer type		-	-
	Invasive lobular carcinoma	140	46.7%
	Invasive ductal carcinoma^d^	117	39.0%
	Ductal carcinoma in situ	37	12.3%
	Lobular carcinoma in situ	3	1.0%
	Inflammatory breast cancer	2	0.7%
	Mucinous carcinoma	1	0.3%
Cancer stage at diagnosis^c^			
	1	26	8.7%
	2	186	62.0%
	3	81	27.0%
	4	5	1.7%
	Unknown	2	0.7%
Disease duration (in years)		2.45	3.18
Metastasis		24	8.0%
Current treatment^a^		-	-
	Immunotherapy	253	84.3%
	Chemotherapy	37	12.3%
	Radiation therapy	11	3.7%
	Stem cell or bone marrow	2	0.7%
	Unknown	2	0.7%
	Palliative care	23	7.7%
Surgery history^f^		243	81.0%
Number of comorbidities^g^			
	0	78	26.0%
	1	123	41.0%
	2+	99	33.0%
Number of symptoms in the past week^h^			
	0	17	5.7%
	1–3	71	23.7%
	4–6	68	22.7%
	7–9	60	20.0%
	10+	84	28.0%

^a^May belong in more than one category

^b^IDR= Indonesian Rupiah, 324.34 USD = 5 million IDR (based on the closing 2023 middle exchange rate from Bank Indonesia)

^c^Based on the American Joint Committee on Cancer Staging (0: non-invasive, pre-cancerous, 1: early stage, spread to other tissue in small area, 2: localized, tumor between 20–50 mm and lymph nodes involved or tumor larger than 50 mm with no lymph nodes involved), 3: regional spread, tumor larger than 50 mm with lymph nodes involved in larger region, may have spread to skin or chest wall, 4: metastatic, distant spread beyond the breast and nearby lymph nodes)

^d^Included subtypes: triple negative breast cancer, luminal A, luminal B HER-2 negative, luminal B HER-2 positive, and HER-2 positive

^e^Most common sites were bone (*n* = 7), lung (*n* = 5), and liver (*n* = 3)

^f^Surgeries included single/double mastectomy and lumpectomy

^g^Most common comorbidities: chronic gastritis (*n* = 172), hypertension (*n* = 72), and obesity (*n* = 39)

^h^Most reported symptoms: fatigue (*n* = 175), dizziness (*n* = 143), muscle pain (*n* = 133), sleep problem (123), anxiety (*n* = 122), and hair loss (*n* = 120)

### Financial toxicity, health, and well-being

The majority of patients reported overall good health status with mean EQ-5D-5L index value of 0.85 ± 0.21, mean EQ VAS of 81.18 ± 15.63, and mean EQ-HWB-S index value of 0.84 ± 0.17 (Table 2). The mean FACIT-COST total score was 24.24 ± 8.65. High SFT as measured by the FACIT-COST (≤ 17.5), was experienced by 21% patients (Table 3). Meanwhile, OFT was experienced by 51% patients who reported at least one financial strategy used to cope with their breast cancer treatment. The two most common strategies used by the patients were borrowing from relatives or financial institution (30.0%) and withdrawing from savings/pension (25.7%).

**Table 2:** Descriptive statistics of the outcome measures

Measure	Theoretical range	Observed range	Mean	Standard deviation	Q1	Median	Q3
FACIT-COST total score^a, e^	0–44	2–42	24.24	8.65	19	25	30
EQ-5D-5L index value^a, b^	-0.865 to 1	-0.31 to 1	0.85	0.21	0.80	0.91	1
EQ VAS^a^	0–100	10–100	81.18	15.63	75	80	90
EQ-HWB-S index value^a, d^	-0.384 to 1	-0.245 to 1	0.84	0.17	0.79	0.89	0.95
EQ-HWB LSS^c^	0–100	0–65	16.48	11.76	8	13	23

Abbreviations. EQ-HWB = EQ Health and Wellbeing, EQ-HWB-S = EQ-HWB short form, EQ VAS = EQ Visual analogue scale, FACIT-COST = COST - A FACIT Measure of Financial Toxicity, LSS = level summary scores

^a^Higher scores indicate better health-related quality of life, better health and well-being, or lower financial toxicity

^b^Computed using the Indonesian value set (Purba et al., 2017)

^c^LSS recoded into a 0-100 scale, with higher scores indicating worse health and well-being

^d^Computed using the pilot UK value set (Mukuria et al., 2023)

^e^Following the scoring guidelines, the 12th item of FACIT-COST was not included in the overall score computation

**Table 3:** EQ-5D-5L, EQ VAS, and EQ-HWB scores across financial toxicity categories

Financial toxicity		*n*	%	Mean EQ-5D-5L index value	*p*-value	Mean EQ VAS	*p*-value	Mean EQ-HWB-S index value	*p*-value			Mean EQ-HWB LSS^a^	*p*-value
Subjective financial toxicity (SFT)^b, c^													
	High SFT	63	21.0%	0.75 ± 0.23	< 0.001	72.94 ± 17.75	< 0.001	0.73 ± 0.24	< 0.001			24.44 ± 14.65	< 0.001
	Low SFT	237	79.0%	0.87 ± 0.19		83.38 ± 14.28		0.87 ± 0.13				14.36 ± 9.87	
Objective financial toxicity (OFT)^d^													
	At least one OFT	153	51.0%	0.82 ± 0.23	0.027	79.74 ± 17.03	0.103	0.82 ± 0.19	0.030			17.61 ± 11.95	0.089
	No OFT	147	49.0%	0.87 ± 0.17		82.69 ± 13.93		0.86 ± 0.14				15.30 ± 11.48	
	*Borrowing from relatives or financial institution*												
	-Yes	90	30.0%	0.81 ± 0.21	0.061	78.39 ± 16.62	0.042	0.79 ± 0.21	0.002			19.30 ± 13.15	0.006
	-No	210	70.0%	0.86 ± 0.20		82.38 ± 15.07		0.86 ± 0.15				15.27 ± 10.92	
	*Withdrawing savings or pension*												
	-Yes	77	25.7%	0.82 ± 0.26	0.185	79.94 ± 15.95	0.417	0.82 ± 0.20	0.320			17.13 ± 12.43	0.573
	-No	223	74.3%	0.85 ± 0.19		81.61 ± 15.53		0.85 ± 0.16				16.25 ± 11.54	
	*Selling assets (e.g., vehicle, land)*												
	-Yes	33	11.0%	0.76 ± 0.25	0.010	75.76 ± 18.38	0.034	0.75 ± 0.26	< 0.001			22.70 ± 14.85	0.001
	-No	267	89.0%	0.86 ± 0.20		81.85 ± 15.16		0.85 ± 0.15				15.71 ± 11.12	
	*Closing business*												
	-Yes	10	3.3%	0.78 ± 0.25	0.270	78.50 ± 12.92	0.582	0.76 ± 0.13	0.142			22.40 ± 12.12	0.105
	-No	290	96.7%	0.85 ± 0.20		81.28 ± 15.73		0.84 ± 0.17				16.27 ± 11.72	
SFT and OFT													
	High SFT and at least one OFT	43	14.3%	0.73 ± 0.25	< 0.001	69.30 ± 17.48	< 0.001	0.71 ± 0.25	< 0.001			24.60 ± 14.18	< 0.001
	High SFT and no OFT	20	6.7%	0.81 ± 0.17		80.75 ± 16.08		0.77 ± 0.22				24.10 ± 15.99	
	Low SFT and at least one OFT	110	36.7%	0.86 ± 0.21		83.82 ± 15.07		0.86 ± 0.14				14.87 ± 9.74	
	Low SFT and no OFT	127	42.3%	0.88 ± 0.17		82.99 ± 13.60		0.88 ± 0.13				13.91 ± 10.00	
FACIT-COST item 12^e^													
	Quite a bit/very much	85	28.3%	0.79 ± 0.21	< 0.001	74.82 ± 17.12	< 0.001	0.76 ± 0.22	< 0.001			22.93 ± 13.77	< 0.001
	A little bit/somewhat	114	38.0%	0.84 ± 0.24		81.93 ± 15.66		0.84 ± 0.16				17.07 ± 10.77	
	Not at all	101	33.7%	0.90 ± 0.12		85.69 ± 12.31		0.91 ± 0.09				10.38 ± 7.04	

Abbreviations. EQ-HWB = EQ Health and Wellbeing, EQ-HWB-S = EQ-HWB short form, FACIT-COST = COST - A FACIT Measure of Financial Toxicity, LSS = level summary scores

^a^LSS recoded to a 0-100 scale

^b^High subjective financial toxicity: FACIT-COST score of ≤ 17.5 (Ng et al., 2021)

^c^Following the scoring guidelines, item 12 of the FACIT-COST was not included in the overall score computation

^d^Each patient may have incurred more than one financial coping strategy

^e^’Financial hardship to my family and me’ item (responses recoded from five to three levels)

**Fig. 1:**
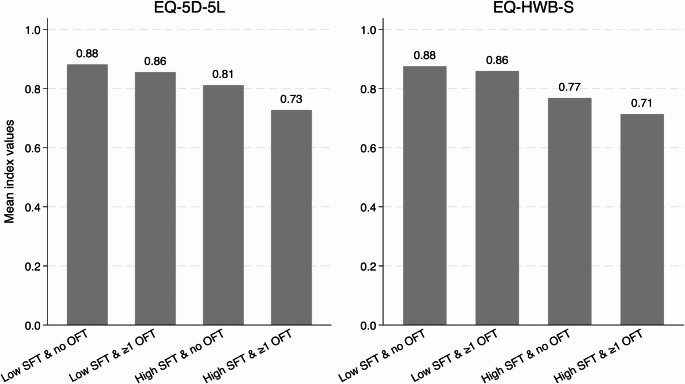
Mean EQ-5D-L and EQ-HWB-S index values across financial toxicity subgroups. *Abbreviations.* EQ-HWB-S: EQ Health and Wellbeing short form, OFT: objective financial toxicity, SFT: subjective financial toxicity

Among the four coping strategies, patients who sold their assets had the lowest mean EQ-5D-5L and EQ-HWB-S index values of 0.76 ± 0.25 and 0.75 ± 0.26, respectively. Overall, 42.3% experienced low SFT and no OFT, 36.7% experienced low SFT but at least one OFT, 6.7% experienced high SFT and no OFT, and 14.3% experienced both high SFT and at least one OFT. The mean EQ-5D-5L index values for these four subgroups were 0.88 ± 0.17, 0.86 ± 0.21, 0.81 ± 0.17, 0.73 ± 0.25, while the mean EQ-HWB-S index values were 0.88 ± 0.13, 0.86 ± 0.14, 0.77 ± 0.22, and 0.71 ± 0.25 respectively (*p* < 0.001 for both instruments) (Fig. 1). The EQ-5D-L and EQ-HWB-S index values had statistically significant differences for the FACIT-COST item ‘financial hardship to my family and me’: not at all (0.90 ± 0.12, 0.91 ± 0.09), a little bit/somewhat (0.84 ± 0.24, 0.84 ± 0.16), and quite a bit/very much (0.79 ± 0.21, 0.76 ± 0.22) (*p* < 0.001). Comparisons of EQ-5D-5L and EQ-HWB index values or scores among subgroups as defined by socio-demographic and clinical characteristics are presented in Supplementary Material 1.

### Correlations between FACIT-COST, EQ-5D-5L, and EQ-HWB

The FACIT-COST total score demonstrated correlations that were borderline moderate with EQ-HWB coping (-0.34), EQ-HWB-S no control over daily life (-0.33), exhaustion (-0.31), and weakly correlated with the following items: EQ-HWB frustration (-0.29), EQ-HWB-S sadness/depression (-0.28), EQ-5D-5L pain/discomfort (-0.28), and anxiety/depression (-0.27), among others (Table 4). At the instrument level, FACIT-COST total score exhibited moderate correlations with EQ-HWB LSS (-0.48), EQ-HWB-S index values (0.44), EQ VAS scores (0.44), EQ-5D-5L LSS (-0.32), and EQ-5D-5L index values (0.30).

**Table 4:** Correlations between the EQ-5D-5L, EQ-HWB, and FACIT-COST

	FACIT-COST total score^*^
***Pearson’s correlations***	
EQ-5D-5L index value	0.30
EQ VAS	0.35
EQ-HWB-S index value	0.44
EQ-HWB LSS	-0.48
***Spearman’s correlations***	
EQ-5D-5L pain/discomfort	-0.28
EQ-5D-5L anxiety/depression	-0.27
EQ-HWB-S exhaustion	-0.31
EQ-HWB-S anxiety	-0.22
EQ-HWB-S sadness/depression	-0.28
EQ-HWB-S pain (severity)	-0.23
EQ-HWB-S no control over daily life	-0.33
EQ-HWB frustration	-0.29
EQ-HWB coping	-0.34
EQ-HWB discomfort (severity)	-0.19

Abbreviations. EQ-HWB = EQ Health and Wellbeing, EQ-HWB-S = EQ-HWB short form, FACIT-COST = COST - A FACIT Measure of Financial Toxicity, LSS = level summary scores

^*^Following the scoring guidelines, the 12th item of FACIT-COST was not included in the overall score computation

All correlation coefficients were *p* < 0.001

### Associations between financial toxicity and EQ-5D-5L and EQ-HWB items

After adjusting for socio-demographic and clinical covariates, reporting higher SFT was associated with more problems in the EQ-5D-5L pain/discomfort (OR = 1.07), anxiety/depression (OR = 1.06), EQ-HWB-S exhaustion (OR = 1.06), anxiety (OR = 1.04), sadness/depression (OR = 1.06), pain (OR = 1.06), EQ-HWB frustration (OR = 1.10), and discomfort (OR = 1.04) items (Table 5). Meanwhile, higher OFT was only significantly associated with more problems in the EQ-HWB-S exhaustion item (OR = 1.40).

**Table 5:** Ordinal logistic regression results

Variables	EQ-5D-5L pain/discomfort		EQ-5D-5L anxiety/depression		EQ-HWB-S exhaustion		EQ-HWB-S anxiety		EQ-HWB-S sadness/depression		EQ-HWB-S pain (severity)		EQ-HWB frustration		EQ-HWB discomfort (severity)	
	OR	95% CI	OR	95% CI	OR	95% CI	OR	95% CI	OR	95% CI	OR	95% CI	OR	95% CI	OR	95% CI
Subjective financial toxicity^a^	1.07^***^	(1.04, 1.1)	1.06^**^	(1.02, 1.10)	1.06^***^	(1.03, 1.10)	1.04^**^	(1.01, 1.08)	1.06^**^	(1.02, 1.09)	1.06^***^	(1.03, 1.10)	1.10^**^	(1.05, 1.15)	1.04^*^	(1.01, 1.07)
Objective financial toxicity	1.16	(0.87, 1.56)	1.23	(0.88, 1.71)	1.40^*^	(1.06, 1.87)	1.12	(0.84, 1.51)	1.18	(0.87, 1.59)	0.94	(0.71, 1.24)	0.90	(0.61, 1.34)	1.03	(0.77, 1.36)
Pseudo R-squared	12.63%		14.40%		15.14%		9.68%		6.68%		9.20%		17.34%		10.34%	

^***^p < 0.001, ^**^p < 0.01, ^*^p < 0.05

Abbreviations. CI = confidence interval, EQ-HWB = EQ Health and Wellbeing, EQ-HWB-S = EQ-HWB short form, OR = odds ratio

All regression models were controlled for age, income, number of children, diagnosis duration, metastasis status, current chemotherapy, number of comorbidities and symptoms in the past week

^a^Measured using COST - A FACIT Measure of Financial Toxicity

### Associations between financial toxicity and EQ-5D-5L and EQ-HWB level sum scores and index values

In the unadjusted OLS models, higher SFT was significantly associated with lower EQ-5D-5L index value (‘Model 1’), EQ VAS (‘Model 4’), EQ-HWB-S index value (‘Model 7’), and higher EQ-HWB LSS (‘Model 10’) (*p* < 0.001 each) (Table 6). After controlling for the socio-demographic and clinical covariates, the significant associations between SFT and the outcomes persisted (*p* < 0.001 each): EQ-5D-5L index value (beta=-0.01, ‘Model 3’), EQ VAS (beta=-0.56, ‘Model 6’), EQ-HWB-S index value (beta=-0.01, ‘Model 9’), and EQ-HWB LSS (beta = 0.54, ‘Model 12’). After covariate adjustment, FT explained more variance in EQ-HWB-S index value (R^2^ = 46.39%) and EQ-HWB LSS (R^2^ = 46.15%) than in EQ-5D-5L index value (R^2^ = 31.23%) and EQ VAS (R^2^ = 25.60%).

**Table 6:** Multivariable linear regression results

Variables		Outcome: EQ-5D-5L index value						Outcome: EQ VAS					
		Model 1		Model 2		Model 3		Model 4		Model 5		Model 6	
		B	SE	B	SE	B	SE	B	SE	B	SE	B	SE
*Intercept*		*1.00*	*0.00*	0.98	0.05	1.13	0.03	95.15	1.98	98.08	2.65	99.94	3.75
Subjective financial toxicity^a^		-0.01^***^	0.01	-0.01^***^	0.00	-0.01^***^	0.00	-0.63^***^	0.11	-0.66^***^	0.11	-0.56^***^	0.11
Objective financial toxicity		-0.02	0.03	-0.03	0.01	-0.02	0.01	-0.49	0.97	-0.87	0.96	-0.22	0.94
Aged 50 years and above		-	-	-0.06^**^	0.02	-0.03	0.02	-	-	-5.26^**^	1.67	-3.53^*^	1.69
Income > 5 million IDR^b^		-	-	-0.08	0.05	-0.01^*^	0.05	-	-	1.64	1.96	0.57	1.88
Number of children		-	-	0.02^*^	0.01	0.03^**^	0.01	-	-	1.11	0.77	1.25	0.79
Diagnosed 1 year or less		-	-	-	-	-0.04	0.02	-	-	-	-	-0.29	1.68
Metastasis		-	-	-	-	-0.11^*^	0.05	-	-	-	-	1.74	3.08
Undergoing chemotherapy		-	-	-	-	-0.10^**^	0.04	-	-	-	-	-5.12	2.94
Comorbidities *(ref: none)*													
	1	-	-	-	-	-0.01	0.03	-	-	-	-	-1.77	1.82
	2+	-	-	-	-	-0.03	0.03	-	-	-	-	-3.89	2.31
Symptoms in the past week *(ref: none)*													
	1–3	-	-	-	-	-0.02	0.03	-	-	-	-	2.49	3.22
	4–6	-	-	-	-	-0.07^*^	0.03	-	-	-	-	-2.36	3.36
	7–9	-	-	-	-	-0.09^**^	0.03	-	-	-	-	-2.32	3.53
	10+	-	-	-	-	-0.18^***^	0.04	-	-	-	-	-8.77^*^	3.55
Model fit		F(2,297) = 16.01 (*p* < 0.001) R^2^ = 9.12%		F(5,294) = 8.11 (*p* < 0.001) R^2^ = 14.88%		F(14,285) = 7.50 (*p* < 0.001) R^2^ = 31.23%		F(2,297) = 21.37 (*p* < 0.001) R^2^ = 12.63%		F(5,294) = 11.50 (*p* < 0.001) R^2^ = 16.36%		F(14,285) = 6.27 (*p* < 0.001) R^2^ = 25.60%	
**Variables**		**Outcome: EQ-HWB-S index value**						**Outcome: EQ-HWB LSS** ^**c**^					
		**Model 7**		**Model 8**		**Model 9**		**Model 10**		**Model 11**		**Model 12**	
		***B***	***SE***	***B***	***SE***	***B***	***SE***	***B***	***SE***	***B***	***SE***	***B***	***SE***
*Intercept*		1.02	0.03	1.07	0.04	1.11	0.04	2.39	1.53	-0.84	2.04	-3.82	2.54
Subjective financial toxicity^a^		-0.01^*^	0.00	-0.01^***^	0.00	-0.01^***^	0.00	0.63^***^	0.08	0.69^***^	0.08	0.54^***^	0.07
Objective financial toxicity		-0.02	0.01	-0.02	0.01	-0.01	0.01	0.61	0.84	1.00	0.82	-0.03	0.73
Aged 50 years and above		-	-	-0.06^**^	0.02	-0.03	0.02	-	-	3.81^**^	1.28	1.88	1.16
Income > 5 million IDR^b^		-	-	-0.05^*^	0.03	-0.07^**^	0.02	-	-	2.50	1.69	3.27^*^	1.45
Number of children		-	-	0.02^**^	0.01	0.02^**^	0.01	-	-	-0.98	0.58	-0.68	0.56
Diagnosed 1 year or less		-	-	-	-	0.00	0.02	-	-	-	-	-1.58	1.06
Metastasis		-	-	-	-	-0.09^*^	0.04	-	-	-	-	4.83	2.76
Undergoing chemotherapy		-	-	-	-	-0.06^*^	0.03	-	-	-	-	1.62	1.65
Comorbidities *(ref: none)*													
	1	-	-	-	-	-0.01	0.02	-	-	-	-	1.96	1.23
	2+	-	-	-	-	-0.03	0.02	-	-	-	-	2.13	1.40
Symptoms in the past week *(ref: none)*													
	1–3	-	-	-	-	-0.01	0.02	-	-	-	-	1.28	2.13
	4–6	-	-	-	-	-0.05^*^	0.02	-	-	-	-	4.26^*^	2.12
	7–9	-	-	-	-	-0.05	0.03	-	-	-	-	5.98^**^	2.28
	10+	-	-	-	-	-0.18^***^	0.03	-	-	-	-	14.06^***^	2.25
Model fit		F(2,297) = 19.94 (*p* < 0.001) R^2^ = 19.60%		F(5,294) = 9.71 (*p* < 0.001) R^2^ = 25.98%		F(14,285) = 8.82 (*p* < 0.001) R^2^ = 46.39%		F(2,297) = 34.21 (*p* < 0.001) R^2^ = 22.74%		F(5,294) = 16.58 (*p* < 0.001) R^2^ = 26.94%		F(14,285) = 15.31 (*p* < 0.001) R^2^ = 46.15%	

^***^p < 0.001, ^**^p < 0.01, ^*^p < 0.05

*Abbreviations. B = unstandardized beta coefficient, EQ HWB: EQ Health and Wellbeing, EQ-HWB-S: EQ-HWB short form, LSS = level summary scores, SE = robust standard error of the regression*

^a^Measured using COST - A FACIT Measure of Financial Toxicity

^b^Net monthly household income. IDR = Indonesian Rupiah, 324.34 USD = 5 million IDR (based on the closing 2023 middle exchange rate from Bank Indonesia)

^c^LSS recoded to a 0-100 scale, with higher scores indicating worse health and well-being

## Discussion

This study aimed to examine the associations between FT, HRQoL, and well-being outcomes in patients with breast cancer. We demonstrated higher SFT to be associated with more problems in EQ-5D-5L pain/discomfort, anxiety/depression, EQ-HWB-S exhaustion, anxiety, sadness/depression, pain, EQ-HWB frustration, discomfort items, lower EQ-5D-5L index value, EQ VAS, EQ-HWB-S index value, and higher EQ-HWB LSS. Higher OFT was also related to more problems in the EQ-HWB-S exhaustion item.

The distress brought about by the financial challenges arising from cancer care was, to some extent, captured by the EQ-5D-5L, EQ VAS, and EQ-HWB. This could be attributed to increased negative emotions related to financial difficulties. Insufficient financial resources may hinder access to optimal healthcare, potentially leading to a diminished HRQoL and well-being [55, 56]. Alternatively, it is also possible that the association is bi-directional as shown by studies using HRQoL to predict SFT [15]. It can be argued that patients with worse HRQoL or well-being subjectively report higher FT due to their condition and possible productivity loss. Hence, complementing the measurement of SFT with OFT seems important for a more comprehensive description of FT by identifying financial metrics or activities of patients.

Our findings suggest that FT accounted for a greater proportion of the variances in well-being, compared to HRQoL. Higher FT could mean that patients may have to make sacrifices in terms of necessities and wants, which may be related to feelings of isolation and frustration. Well-being may better capture the dynamics of FT, as it may include domains broader than HRQoL, such as pursuits that individuals desire or find meaningful, and sense of connection with one’s environment.

Overall, our results align with the existing literature from other countries and neighboring regions. Previous studies conducted in the United States, Australia, and China, focusing on various cancer types such as gastrointestinal, gynecological, and lung, have investigated associations between the SFT (FACIT-COST) and HRQoL as measured by the EQ-5D; employing other diverse methods such as generalized linear model, latent class analysis, and correlations [52, 57–60]. All the studies demonstrated SFT to be significantly related to lower HRQoL. Additionally, two studies, found SFT to be moderately correlated with well-being [25, 61]. Recent studies have also demonstrated significant associations between FT and EQ-5D-5L pain/discomfort and anxiety/depression domains with comparable association strengths [50–52], suggesting that FT captures or represents a form of psychological distress, a burden commonly experienced by patients with cancer. Patients with higher symptom burden may experience greater financial strain due to non-medical costs related to symptom management and hospital visits, intensifying their psychological distress.

Our analysis did not reveal a statistically significant association between OFT and the outcome variable across most regression models, despite showing significance in the subgroup comparisons. This suggests that the OFT measurement may have benefitted from a more comprehensive approach, such as the currency amount of out-of-pocket health expenditure, as well as more detailed exploration of the financial coping strategies (e.g., loan amount or receipt from sale of assets). For example, two investigations from China and Malaysia found negative associations between both SFT and OFT with HRQoL [48, 62, 63]. Notably, these two studies consistently measured OFT using the healthcare cost-to-income ratio, while HRQoL was assessed using various instruments: EORTC QLQ-C30, EQ-5D-5L, and FACT-Lung. However, obtaining precise data on actual healthcare costs may present challenges, such as the patient not being completely in charge of their own finances. Recalling the accurate cost amount would also be challenging, particularly in the case of our sample, whose average disease duration since diagnosis was 2.45 years and nearly 100% had insurance coverage that mitigated direct medical expenses, including diagnostic tests, medications, surgeries, and physician fees.

Reflecting on our findings, some policy implications may be considered. While causality has not been established, our findings indicate a significant correlation between higher FT and diminished HRQoL and well-being. Health and social policymakers may consider interventions aimed at alleviating FT. Firstly, it may be important to screen for FT in patients and their families. Through proper identification of those at risk, necessary mitigation strategies can be implemented. One of the most adopted FT interventions involves financial navigation programs aimed at supporting patients and families with managing the financial hardships of their treatment [64–66]. In the most extreme cases of poverty, extending coverage to include non-medical, cancer-related costs (e.g., transportation and accommodation for outpatients residing at a distance from healthcare facilities) may be an approach. The income-earning capacities of patients should also be protected from disruptions due to cancer [67], such as through employment reintegration programs to facilitate their return to work [68].

This study has some limitations. First, the data were collected from a single center in one country focusing on females with breast cancer. There are also less developed areas in Indonesia with higher poverty rate and lower access to healthcare. Therefore, the results may not be generalized to other types of cancer, male patients, or more resource-poor settings. Second, we solely focused on patients and did not include their caregivers or core family members. In the Indonesian context, men are still predominantly perceived as providers. Our sample primarily consisted of female homemakers and thus, FT may not have been comprehensively captured without the perspectives of the income provider. Third, nearly all patients had insurance coverage that may have led to some socio-demographic covariates not being significantly associated with the outcome variables and excluded from the regressions. However, this could also be attributed to limited response variability. Fourth, our measurement of SFT had its drawbacks. The FACIT-COST was developed in the United States and another measure may be more suited to capture financial well-being in the Indonesian context. However, it is the most widely used cancer-specific measure for SFT, allowing for comparability with previous studies. Fifth, the pilot UK value set was used for calculating the EQ-HWB-S index values, which does not fully reflect the preferences of the Indonesian population. Finally, our study design did not allow us to explore causality, which could be examined in future studies along with potential mediating factors, such as social support.

## Conclusions

This is the first study to identify associations between FT, HRQoL, and well-being outcomes in patients with breast cancer, and the first in the FT literature to use the recently developed EQ-HWB instrument to measure health and well-being. Our findings provide additional insight into the burden of cancer and its link to the HRQoL and well-being of patients in a middle-income country context, highlighting the importance of establishing health and social policies aimed at measuring and alleviating FT.

## Electronic supplementary material

Below is the link to the electronic supplementary material.

Supplementary Material 1

## Data Availability

The data that support the results of this study are available from FDP, upon reasonable request.
