# Supplementary material for: Associations between financial toxicity, health-related quality of life, and well-being in Indonesian patients with breast cancer
Source: Qual Life Res. 2025 Feb 25;34(6):1709–22. doi: 10.1007/s11136-025-03925-y (PMC12119780; doi:10.1007/s11136-025-03925-y)
Supplement: Supplementary file 1 — Supplementary Material 1 [file 11136_2025_3925_MOESM1_ESM.docx]

**Electronic Supplementary Material**

**Online Resource 1. Comparisons of EQ-5D-5L index values, EQ VAS scores, EQ-HWB-S index values, and EQ-HWB level sum scores among patient subgroups**

| **Variable** | **Category** | **Financial toxicity subgroup** | **n** | **Mean EQ-5D-5L index value^a^** | **p-value** | **Mean EQ VAS^a^** | **p-value** | **Mean EQ-HWB-S index value^a^** | **p-value** | **Mean EQ-HWB level sum score^b^** | **p-value** |
| --- | --- | --- | --- | --- | --- | --- | --- | --- | --- | --- | --- |
| Age | Aged < 50 | Low SFT | 91 | 0.90 (0.12) | **< 0.001** | 86.09 (12.47) | **< 0.001** | 0.89 (0.10) | **< 0.001** | 12.85 (8.54) | **< 0.001** |
|  |  | High SFT | 41 | 0.79 (0.21) |  | 75.61 (15.74) |  | 0.77 (0.18) |  | 21.90 (12.62) |  |
|  |  | No OFT | 56 | 0.89 (0.13) | 0.138 | 84.82 (13.24) | 0.175 | 0.88 (0.16) | 0.183 | 13.96 (12.30) | 0.138 |
|  |  | With OFT | 76 | 0.85 (0.18) |  | 81.38 (15.05) |  | 0.84 (0.12) |  | 16.79 (9.46) |  |
|  | Aged ≥ 50 | Low SFT | 146 | 0.85 (0.22) | **0.002** | 81.68 (15.09) | **< 0.001** | 0.85 (0.14) | **< 0.001** | 15.36 (10.52) | **< 0.001** |
|  |  | High SFT | 22 | 0.68 (0.25) |  | 67.95 (20.45) |  | 0.65 (0.31) |  | 29.18 (17.16) |  |
|  |  | No OFT | 91 | 0.86 (0.19) | 0.056 | 81.37 (14.24) | 0.203 | 0.85 (0.13) | **0.048** | 16.12 (10.93) | 0.235 |
|  |  | With OFT | 77 | 0.79 (0.27) |  | 78.12 (18.74) |  | 0.80 (0.23) |  | 18.42 (14.00) |  |
| Income | > 5 million IDR | Low SFT | 30 | 0.84 (0.26) | N/A | 87.50 (8.88) | N/A | 0.86 (0.15) | N/A | 13.33 (8.68) | N/A |
|  |  | High SFT | 0 | - |  | - |  | - |  | - |  |
|  |  | No OFT | 22 | 0.81 (0.29) | 0.255 | 87.27 (8.41) | 0.821 | 0.86 (0.16) | 0.603 | 13.95 (8.62) | 0.525 |
|  |  | With OFT | 8 | 0.93 (0.10) |  | 88.13 (10.67) |  | 0.89 (0.12) |  | 11.63 (9.21) |  |
|  | 5 million IDR or less | Low SFT | 207 | 0.87 (0.18) | **< 0.001** | 82.78 (14.82) | **< 0.001** | 0.87 (0.13) | **< 0.001** | 14.51 (10.04) | **< 0.001** |
|  |  | High SFT | 63 | 0.75 (0.23) |  | 72.94 (17.75) |  | 0.73 (0.24) |  | 24.44 (14.65) |  |
|  |  | No OFT | 125 | 0.88 (0.14) | **0.004** | 81.88 (14.56) | 0.185 | 0.86 (0.14) | **0.023** | 15.54 (11.92) | 0.102 |
|  |  | With OFT | 145 | 0.81 (0.23) |  | 79.28 (17.22) |  | 0.81 (0.19) |  | 17.94 (12.02) |  |
| Number of children | None | Low SFT | 116 | 0.84 (0.24) | **0.001** | 82.28 (16.32) | **< 0.001** | 0.85 (0.16) | **< 0.001** | 15.15 (10.49) | **< 0.001** |
|  |  | High SFT | 28 | 0.68 (0.23) |  | 67.68 (18.53) |  | 0.63 (0.28) |  | 30.18 (15.77) |  |
|  |  | No OFT | 76 | 0.86 (0.20) | **0.021** | 81.05 (15.61) | 0.250 | 0.84 (0.17) | **0.038** | 16.20 (12.05) | 0.068 |
|  |  | With OFT | 68 | 0.76 (0.28) |  | 77.65 (19.71) |  | 0.77 (0.24) |  | 20.18 (13.94) |  |
|  | 1 or more child(ren) | Low SFT | 121 | 0.89 (0.13) | **0.006** | 84.42 (11.98) | **0.004** | 0.89 (0.09) | **< 0.001** | 13.59 (9.21) | **0.001** |
|  |  | High SFT | 35 | 0.81 (0.22) |  | 77.14 (16.14) |  | 0.81 (0.16) |  | 19.86 (12.05) |  |
|  |  | No OFT | 71 | 0.89 (0.14) | 0.353 | 84.44 (11.73) | 0.159 | 0.88 (0.11) | 0.186 | 14.33 (10.84) | 0.461 |
|  |  | With OFT | 85 | 0.87 (0.17) |  | 81.41 (14.44) |  | 0.86 (0.12) |  | 15.55 (9.69) |  |
| Diagnosis | 1 year or less | Low SFT | 117 | 0.84 (0.23) | **0.021** | 82.82 (15.37) | **< 0.001** | 0.86 (0.15) | **< 0.001** | 14.54 (9.83) | **< 0.001** |
|  |  | High SFT | 39 | 0.75 (0.21) |  | 71.54 (17.25) |  | 0.75 (0.19) |  | 22.85 (12.26) |  |
|  |  | No OFT | 76 | 0.86 (0.21) | 0.054 | 83.16 (13.49) | **0.020** | 0.86 (0.16) | 0.080 | 14.84 (10.99) | 0.051 |
|  |  | With OFT | 80 | 0.78 (0.25) |  | 77.00 (18.60) |  | 0.81 (0.18) |  | 18.30 (10.92) |  |
|  | 1 + year | Low SFT | 120 | 0.90 (0.14) | **< 0.001** | 83.92 (13.16) | **0.007** | 0.88 (0.10) | **< 0.001** | 14.18 (9.94) | **< 0.001** |
|  |  | High SFT | 24 | 0.77 (0.26) |  | 75.20 (18.68) |  | 0.70 (0.30) |  | 27.04 (17.86) |  |
|  |  | No OFT | 71 | 0.89 (0.13) | 0.266 | 82.18 (14.46) | 0.819 | 0.87 (0.13) | 0.189 | 15.79 (12.04) | 0.613 |
|  |  | With OFT | 73 | 0.86 (0.21) |  | 82.74 (14.67) |  | 0.83 (0.20) |  | 16.85 (13.02) |  |
| Metastasis status | Not metastatic cancer | Low SFT | 220 | 0.88 (0.18) | **< 0.001** | 83.48 (14.19) | **< 0.001** | 0.88 (0.12) | **< 0.001** | 13.92 (9.32) | **< 0.001** |
|  |  | High SFT | 56 | 0.77 (0.21) |  | 73.13 (17.98) |  | 0.76 (0.19) |  | 23.25 (12.96) |  |
|  |  | No OFT | 136 | 0.88 (0.16) | 0.061 | 82.79 (13.87) | 0.136 | 0.87 (0.14) | 0.079 | 14.76 (11.09) | 0.113 |
|  |  | With OFT | 140 | 0.84 (0.20) |  | 80.00 (17.00) |  | 0.84 (0.15) |  | 16.83 (10.47) |  |
|  | Metastatic cancer | Low SFT | 17 | 0.74 (0.30) | 0.354 | 82.06 (15.72) | 0.155 | 0.77 (0.22) | 0.086 | 20.06 (14.51) | 0.090 |
|  |  | High SFT | 7 | 0.60 (0.37) |  | 71.43 (17.01) |  | 0.53 (0.44) |  | 34.00 (23.65) |  |
|  |  | No OFT | 11 | 0.78 (0.18) | 0.266 | 81.36 (15.18) | 0.523 | 0.79 (0.20) | 0.170 | 21.91 (14.49) | 0.596 |
|  |  | With OFT | 13 | 0.63 (0.40) |  | 76.92 (17.86) |  | 0.63 (0.38) |  | 26.00 (21.39) |  |
| Chemotherapy | Not undergoing chemotherapy | Low SFT | 210 | 0.88 (0.17) | **< 0.001** | 83.81 (13.34) | **< 0.001** | 0.88 (0.12) | **< 0.001** | 14.22 (9.69) | **< 0.001** |
|  |  | High SFT | 53 | 0.76 (0.23) |  | 73.40 (18.26) |  | 0.74 (0.21) |  | 24.75 (14.01) |  |
|  |  | No OFT | 124 | 0.88 (0.16) | 0.062 | 82.74 (14.09) | 0.294 | 0.87 (0.14) | 0.075 | 15.27 (11.36) | 0.151 |
|  |  | With OFT | 139 | 0.84 (0.21) |  | 80.79 (15.80) |  | 0.83 (0.16) |  | 17.30 (11.53) |  |
|  | Undergoing chemotherapy | Low SFT | 27 | 0.77 (0.30) | 0.537 | 80.00 (20.14) | 0.186 | 0.82 (0.19) | 0.219 | 15.44 (11.33) | 0.151 |
|  |  | High SFT | 10 | 0.70 (0.25) |  | 70.50 (15.36) |  | 0.70 (0.36) |  | 22.80 (18.57) |  |
|  |  | No OFT | 23 | 0.82 (0.22) | 0.051 | 82.39 (13.30) | **0.043** | 0.84 (0.16) | 0.080 | 15.48 (12.32) | 0.274 |
|  |  | With OFT | 14 | 0.63 (35) |  | 69.29 (24.72) |  | 0.69 (0.33) |  | 20.64 (15.76) |  |
| Number of comorbidities | No comorbidities | Low SFT | 66 | 0.88 (0.25) | 0.463 | 86.59 (13.04) | **0.009** | 0.89 (0.15) | **0.041** | 12.14 (9.82) | **0.011** |
|  |  | High SFT | 12 | 0.82 (0.26) |  | 75.00 (17.96) |  | 0.78 (0.24) |  | 21.33 (17.46) |  |
|  |  | No OFT | 42 | 0.89 (0.24) | 0.613 | 85.36 (11.81) | 0.719 | 0.90 (0.14) | 0.150 | 11.60 (10.08) | 0.110 |
|  |  | With OFT | 36 | 0.86 (0.26) |  | 84.17 (17.09) |  | 0.84 (0.20) |  | 15.83 (13.06) |  |
|  | 1 comorbidity | Low SFT | 95 | 0.82 (0.26) | **< 0.001** | 83.84 (12.97) | **0.011** | 0.89 (0.09) | **< 0.001** | 13.87 (8.59) | **< 0.001** |
|  |  | High SFT | 28 | 0.75 (0.23) |  | 76.43 (14.65) |  | 0.74 (0.18) |  | 24.93 (11.12) |  |
|  |  | No OFT | 62 | 0.88 (0.13) | 0.198 | 83.31 (13.85) | 0.348 | 0.87 (0.13) | 0.222 | 15.61 (11.47) | 0.401 |
|  |  | With OFT | 61 | 0.84 (0.18) |  | 80.98 (13.50) |  | 0.84 (0.13) |  | 17.18 (8.95) |  |
|  | 2 + comorbidities | Low SFT | 76 | 0.83 (0.21) | **0.033** | 80.00 (16.19) | **0.003** | 0.83 (0.15) | **0.006** | 16.89 (10.91) | **0.005** |
|  |  | High SFT | 23 | 0.72 (0.22) |  | 67.61 (20.29) |  | 0.70 (0.29) |  | 25.48 (17.15) |  |
|  |  | No OFT | 43 | 0.85 (0.15) | 0.075 | 79.19 (15.47) | 0.318 | 0.92 (0.16) | 0.394 | 18.47 (11.97) | 0.779 |
|  |  | With OFT | 56 | 0.77 (0.25) |  | 75.54 (19.60) |  | 0.78 (0.23) |  | 19.21 (13.93) |  |
| Number of symptoms | No symptoms | Low SFT | 15 | 0.95 (0.07) | 0.953 | 86.00 (12.28) | 0.661 | 0.94 (0.07) | 0.680 | 8.07 (8.46) | 0.748 |
|  |  | High SFT | 2 | 0.96 (0.06) |  | 90.00 (0.00) |  | 0.96 (0.06) |  | 6.00 (7.07) |  |
|  |  | No OFT | 7 | 0.95 (0.09) | 0.848 | 85.00 (6.45) | 0.675 | 0.95 (0.07) | 0.456 | 5.86 (9.26) | 0.422 |
|  |  | With OFT | 10 | 0.96 (0.05) |  | 87.50 (14.39) |  | 0.93 (0.06) |  | 9.20 (7.44) |  |
|  | 1–3 symptoms | Low SFT | 59 | 0.94 (0.09) | 0.079 | 88.05 (12.22) | 0.971 | 0.93 (0.06) | 0.064 | 9.78 (6.88) | 0.127 |
|  |  | High SFT | 12 | 0.88 (0.16) |  | 87.92 (8.38) |  | 0.88 (0.13) |  | 13.67 (12.11) |  |
|  |  | No OFT | 45 | 0.93 (0.12) | 0.514 | 87.00 (13.29) | 0.330 | 0.92 (0.08) | 0.731 | 10.38 (8.37) | 0.936 |
|  |  | With OFT | 26 | 0.94 (0.07) |  | 89.81 (7.81) |  | 0.92 (0.09) |  | 10.54 (7.54) |  |
|  | 4–6 symptoms | Low SFT | 59 | 0.89 (0.133) | 0.653 | 84.07 (11.28) | 0.298 | 0.89 (0.08) | **0.010** | 12.00 (7.01) | **0.001** |
|  |  | High SFT | 9 | 0.87 (0.14) |  | 79.44 (18.10) |  | 0.80 (0.21) |  | 21.78 (13.15) |  |
|  |  | No OFT | 35 | 0.88 (0.10) | 0.849 | 82.86 (12.14) | 0.683 | 0.88 (0.12) | 0.888 | 13.34 (9.52) | 0.962 |
|  |  | With OFT | 33 | 0.89 (0.16) |  | 84.09 (12.65) |  | 0.88 (0.10) |  | 13.24 (7.70) |  |
|  | 7–9 symptoms | Low SFT | 48 | 0.85 (0.18) | 0.332 | 83.33 (13.85) | **0.006** | 0.87 (0.11) | 0.306 | 15.94 (9.82) | 0.379 |
|  |  | High SFT | 12 | 0.80 (0.14) |  | 70.00 (16.51) |  | 0.83 (0.10) |  | 18.75 (9.84) |  |
|  |  | No OFT | 28 | 0.83 (0.21) | 0.781 | 80.71 (15.79) | 0.982 | 0.85 (0.12) | 0.547 | 17.68 (11.23) | 0.389 |
|  |  | With OFT | 32 | 0.85 (0.13) |  | 80.63 (15.01) |  | 0.87 (0.10) |  | 15.47 (8.41) |  |
|  | 10 + symptoms | Low SFT | 56 | 0.77 (0.29) | **0.035** | 77.05 (17.73) | **0.002** | 0.76 (0.19) | **< 0.001** | 22.00 (10.70) | **< 0.001** |
|  |  | High SFT | 28 | 0.63 (0.26) |  | 64.46 (16.52) |  | 0.59 (0.25) |  | 33.68 (12.60) |  |
|  |  | No OFT | 32 | 0.81 (0.24) | **0.042** | 77.66 (14.76) | 0.058 | 0.74 (0.19) | 0.238 | 24.34 (12.08) | 0.379 |
|  |  | With OFT | 52 | 0.67 (0.31) |  | 69.90 (19.64) |  | 0.68 (0.24) |  | 26.85 (12.90) |  |
| *Abbreviations*. EQ-HWB = EQ Health and Wellbeing, EQ-HWB-S = EQ-HWB short form, EQ VAS = EQ Visual analogue scale, IDR = Indonesian Rupiah, N/A = not applicable, OFT = objective financial toxicity, SFT = subjective financial toxicity  *Note*. Bold p-values indicate p < 0.05  ^a^Higher scores indicate better health-related quality of life or wellbeing  ^b^Higher scores indicate worse wellbeing | | | | | | | | | | | |
